# A qualitative exploration of multi-stakeholder perspectives of before-school physical activity

**DOI:** 10.1186/s12966-024-01572-z

**Published:** 2024-02-29

**Authors:** James Woodforde, Konsita Kuswara, Francisco Perales, Jo Salmon, Sjaan Gomersall, Michalis Stylianou

**Affiliations:** 1 School of Human Movement and Nutrition Sciences, The University of Queensland, St. Lucia, Queensland, 4072 St Lucia, Queensland, Australia; 2 Institute for Physical Activity and Nutrition, School of Exercise and Nutrition Sciences, Deakin University, 3216 Geelong, VIC Australia; 3 School of Social Science, The University of Queensland, 4072 St Lucia, Queensland, Australia; 4 Centre for Health and Wellbeing Research Innovation, School of Human Movement and Nutrition Sciences, The University of Queensland, 4072 St Lucia, Queensland, Australia; 5 School of Health and Rehabilitation Sciences, The University of Queensland, 4072 St Lucia, Queensland, Australia

**Keywords:** Before-school, Physical activity, Influencing factors, School stakeholders, Focus groups

## Abstract

**Background:**

Engagement in before-school physical activity can potentially enhance health and learning-related outcomes for children and adolescents. However, influencing factors and stakeholder perceptions of before-school physical activity remain under-researched. This qualitative study aimed to examine stakeholder perceptions of: *a*) the suitability of the before-school segment for physical activity, *b*) barriers and facilitators associated with before-school physical activity, and *c*) strategies for schools to support before-school physical activity.

**Methods:**

Twelve focus groups and one interview were conducted with 38 participants from a range of school stakeholder groups—students, parents, teachers, school leaders, external physical activity providers, and school health and physical activity experts. Focus groups were analysed using template analysis, guided by a social-ecological model.

**Results:**

Stakeholders perceived before-school physical activity as valuable, for reasons including perceptions of meaningful contributions to students’ cognitive functioning, classroom behaviours, and wellbeing. Factors influencing before-school physical activity were identified across multiple social-ecological levels, including the critical role of school leadership support, availability of facilities, and provision of qualified supervision. Proposed strategies highlighted the need for sustainable design, contextual relevance, and community engagement in before-school initiatives. Additionally, communication of the manifold benefits identified by stakeholders was suggested as a means to drive support and engagement in before-school physical activity.

**Conclusions:**

This study provides insight for schools seeking to enhance opportunities for physical activity in the before-school hours and may inform future intervention research on the subject, taking into account its multi-faceted influences and the need for context-specific strategies.

**Supplementary Information:**

The online version contains supplementary material available at 10.1186/s12966-024-01572-z.

## Introduction

Physical activity (PA) is essential for children and adolescents’ health and development [1]. Growing research suggests that regular PA may also help support academic performance and cognitive function in young people [2, 3]. However, up to two-thirds of children and adolescents do not meet PA guidelines for health benefits [4]. Schools play an important role in supporting PA within this population and can provide students with regular PA opportunities as part of their school routine [5, 6]. 

The World Health Organization recommends the implementation of whole-of-school PA programs in all pre-primary, primary and secondary schools [6]. Various models can guide this approach, including the widely advocated comprehensive school PA program model wherein schools promote PA before school, including through active transport or school-based initiatives, in addition to during and after school [7]. By supporting before-school initiatives, schools expand the range of opportunities available to students to meet PA guidelines [8]. While evidence on school-based before-school PA programs is limited, positive associations with PA outcomes have been synthesised through systematic review [9]. In addition, before-school PA has been shown to enhance on-task behaviour and concentration in the classroom [10, 11]. However, PA-supportive practices and PA levels before school are generally lower than those observed during other parts of the day [12, 13]. 

While work has begun to explore the effectiveness of before-school PA programs, there remains a significant gap in understanding the factors that influence stakeholders’ support and students’ participation during this specific segment of the day. Stakeholder perspectives can enrich our understanding of these influencing factors, as well as the perceived promise and value of the before-school PA segment, thereby contributing to a knowledge base that can inform the planning and development of future initiatives. This takes on added significance given the unique constraints of the before-school segment, which is limited in duration [14] and busy for school staff and families as they prepare for the day [15]. Existing qualitative work on before-school PA is limited, and has typically examined specific programs or focused on single-stakeholder groups [16, 17]. One study indicated that teachers viewed before-school running and walking clubs positively, while acknowledging the lack of student perspectives as a limitation [16]. Another study examined parent perspectives of a before-school multi-activity program, identifying transport, inclement weather and time challenges associated with early mornings as barriers to program participation; however, this study also only considered the perspectives of one stakeholder group [17]. Different stakeholders who are involved in and/or influence PA, including students, teachers, parents, and administrators, may have differing perspectives and experiences; hence, considering multiple perspectives may yield a more comprehensive understanding of aspects relevant to before-school PA.

In the context of these potentially diverse and multifaceted aspects, employing a guiding framework can facilitate a structured and comprehensive exploration of before-school PA. Social-ecological models consider how individual, interpersonal, institutional, community and public policy factors interact and influence health behaviours [18]. These models are widely used in PA research for school-aged children and adolescents and have identified influencing factors at all levels [19, 20]. The use of McLeroy et al’s social-ecological framework in this study allows investigation of the various interplaying factors influencing before-school PA [18, 20]. Consideration of qualitative perspectives across multiple levels of influence can inform the development of effective interventions and resources to support before-school PA among children and adolescents. Therefore, to address the lack of qualitative evidence relating to the segment, this study aimed to examine stakeholder perceptions of: *a*) the suitability of the before-school segment for PA, *b*) influencing factors (barriers and facilitators) associated with before-school PA, and *c*) strategies for schools to support before-school PA.

## Methods

For this study, we have taken a ‘subtle realist’ approach [21]. Ontologically, our position acknowledges the existence of phenomena related to before-school PA independent of the researcher– such as its scheduling, and potential health impacts. However, as we are interested in how before-school PA is perceived by individuals, we also acknowledge that these phenomena are experienced within diverse personal and social contexts, giving rise to a variety of individual experiences. Epistemologically, therefore, we lean towards a relativist position. Through this perspective, we recognise that while there is a reality that exists beyond our individual interpretations, our understanding and knowledge of this reality are inherently influenced by various factors, including the researchers’ and participants’ experiences, backgrounds, and social contexts. Accordingly, we aim to represent valid accounts and ‘common realities’ of before-school PA with reasonable confidence, while acknowledging that other perspectives on the subject also exist [21]. The lead researcher’s background in the teaching profession and prior research experience in the area of before-school PA can be considered relevant factors that affect our representation of accounts, specifically in the planning and facilitation of focus group discussions and interpretation of data.

The study was approved by The University of Queensland’s human research ethics committee [2021/HE000830] in accordance with the National Statement on Ethical Conduct in Human Research.

### Participants

The stakeholder groups eligible for participation in this study were: school students aged 11–17 years (covering both upper primary and secondary school age groups, selected for their developmental stage where they can effectively participate in focus groups), parents of current school students, teachers and principals, external providers (i.e., those who deliver PA services within schools, such as commercial sports coaching), and other individuals with experience and expertise in school PA and health leadership. These participant groups represent key stakeholders involved in facilitating or participating in school PA. Prior experience with before-school PA opportunities was not required for participation in this study. Participants were recruited through a combination of social media and newsletter advertising, and by contacting organisations (e.g., parents’ associations, teacher professional associations) and eligible participants already known to the researchers (e.g., teachers who are alumni of the university’s physical education teacher education program). Upon expressing interest in the study, prospective participants were provided with an information sheet and consent form and invited to provide written consent. In the case of prospective child and adolescent participants, parents or caregivers were provided with an information sheet and consent form and the students were given an assent form.

### Data collection

Data collection took place between December 2021 and December 2022. Data collection primarily occurred through focus groups conducted online via Zoom. However, due to scheduling challenges, one session was held as an individual online interview. Focus groups were chosen as the primary method of data collection to efficiently capture the perspectives of a wide range of participants and promote group interactions in which shared experiences and diverse viewpoints can be explored [22]. Focus group sessions were homogenised by stakeholder group and facilitated by two researchers (JW and either MS or SG). At the time of data collection, the lead facilitator (JW) was a PhD candidate who underwent training in qualitative research methods, and who received ongoing support and training by the co-moderators (MS and SG), who both have experience in qualitative research and conducting focus groups. The lead facilitator had limited contact by email with participants prior to focus groups, except for two teachers with whom he had prior professional relationships.

Focus groups continued until researchers considered the dataset sufficiently rich to carry out the study’s aims. This follows the concept of information power, which suggests that the more study-specific information a sample holds, the fewer participants are needed [23]. In our study, information power was supported by our targeted focus on a specific segment of physical activity, recruitment of multiple groups of stakeholders with relevant and varied experiences, use of established theory, and the high quality of dialogue with and between participants [23]. 

Data collection followed semi-structured guides (developed by JW and revised by the research team), adapted for each stakeholder group. This method was chosen to grant a degree of control and flexibility to participants and enable them to elaborate on their opinions, ideas and attitudes, while maintaining consistency across stakeholder groups and addressing the study’s aims [22]. Guides included open-ended questions on discussion topics such as participants’ experiences with before-school PA, favourable and challenging characteristics of that time period for PA (with prompts related to different social-ecological levels, used when required), and potential strategies for supporting before-school PA. Supplementary File 1 includes a summary of the semi-structured guides. Before focus groups, teachers completed a short survey about their professional context, and parents described their children’s involvement with before-school PA.

### Data analysis

Focus groups and interviews were audio recorded and transcribed verbatim by the lead researcher. Data were analysed using template analysis, a ‘codebook’ thematic analysis technique situated in the ‘middle ground’ between top-down and bottom-up approaches [24]. Template analysis uses iterative coding to identify evidence for themes, organised hierarchically in a template to guide the process [25]. In template analysis, themes are conceptualised as domain summaries [26], and will hereafter be referred to as domains. Template analysis was selected as it provides a balance between structure and flexibility [24], which was suitable for our multiple aims. This combination allowed for coding of concrete, descriptive data (i.e., barriers, facilitators, and strategies) in addition to more open perspectives about the before-school segment requiring more interpretive analysis.

Two *a priori* domains were developed to align with the study’s descriptive aims (*b* and *c*). These were ‘factors influencing before-school PA’ and ‘strategies to support before-school PA’. We used both inductive and deductive coding to establish the template’s hierarchy. Specific influencing factors were first identified through a process of inductive coding, which were then coded deductively to the domain and the social-ecological level which the factor was determined to relate to (*individual*, *family*, *peers*, *school*, *community environment*, *policy*– definitions in Supplementary File 2). Similarly, strategies to support before-school PA were inductively coded for the specific strategies and deductively coded to the domain, before being grouped into like-categories post-analysis. Stakeholder perspectives about the suitability of the segment for PA (aim *a*) were coded inductively to capture all potentially relevant data.

The following steps were implemented to analyse the data transcripts [24]. First, the lead researcher read all transcripts to become familiar with the data and confirm accuracy of transcription. Preliminary coding was then completed by the same researcher on a subset of the data (i.e., one teacher and one parent focus group) using NVivo Mac [27]. This resulted in the production of the initial template, which was refined following a team meeting. From this initial template, a subset of transcripts (one principal and one parent focus group) were independently coded by the lead researcher and another researcher (KK), followed by a debrief meeting. The lead researcher coded remaining transcripts, subsequently revising the template where required to accommodate relevant data that did not align with existing codes. The final coding template is available in Supplementary File 3.

For quality assurance and trustworthiness of our findings, we maintained an audit trail of coding template iterations and changes. This is in addition to our quality checking process of independent coding of a data subset to critically compare coding, identify discrepancies and resolve disagreements through discussion [28]. 

## Results

Twelve focus groups and one interview were conducted with 38 school stakeholders (11 students, 10 parents, 7 teachers, 4 principals, 4 external providers, and 2 school health and PA experts). Participants spanned six states and territories of Australia, mostly residing in Queensland (*n* = 28), followed by Western Australia (*n* = 3), Australian Capital Territory (*n* = 2), New South Wales (*n* = 2), Victoria (*n* = 2), and the Northern Territory (*n* = 1). Both school health and PA experts were trained teachers (one with primary school experience, and one with primary and secondary school experience) who have held roles in health promotion and educational development, focusing on implementing PA strategies and behaviour change programs within schools. External providers had prior experience with before-school PA programming. Detailed characteristics of the sample are presented in Table 1. Focus groups lasted 31–60 min with 2–5 participants in each. The interview lasted 33 min.

**Table 1:** Participant characteristics

**Students** (***n*** **= 11; 3 focus groups)**	
Gender (% girls)	45%
Age (mean, range)	13, 11–15
School level (% secondary school)	55%
**Parents (*n*** **= 10; 3 focus groups)**	
Gender (% women)	100%
Child/ren’s school level	
% primary school	60%
% primary and secondary school	40%
Child has experience with before-school PA programs (% yes)	70%
**Teachers (*n*** **= 7; 2 focus groups)**	
Gender (% women)	71%
School level (% secondary school)	71%
Teaching area (% health and physical education)	71%
Years teaching experience (mean, range)	11, < 1–40
School ICSEA (mean, range)	1056, 960–1138
School remoteness area	
Major city	57%
Inner regional	29%
Remote	14%
Professional experience with before-school PA (% yes)	86%
**Principals (*n*** **= 4; 2 focus groups)**	
Gender (% women)	25%
School level (% secondary school)	75%
Years leadership experience (mean, range)	21, 7–32
**External providers (*n*** **= 4; 1 focus group and 1 interview)**	
Gender (% women)	50%
**School health and PA experts (*n*** **= 2; 1 focus group)**	
Gender (% women)	100%

ICSEA = Index of Community Socio-Educational Advantage, indicates the average level of educational advantage of a school’s student population (standardised average of 1000); PA = physical activity.

Three domains addressing the research questions were identified from the data, including the two *a priori* domains. We summarise these domains with selected illustrative quotes below. All domains and encompassed subdomains alongside supporting quotes are presented in Table 2.

**Table 2:** Summary of domains

		Students	Parents	School staff	External providers	Experts
Domains and subdomains	Example quotes					
**Domain 1: Perceived value of before-school physical activity**						
Cognitive, behavioural and wellbeing impacts	Pa7 (primary school): *I’ve noticed the main benefit is their behaviour. They seem to be a lot better behaved after some physical activity…for my eight year old, his ability to sit down and focus is improved.*	✓	✓	✓	✓	✓
Relationship impacts	EP3: *It is the perfect place. The schools are underutilised for physical activity and movement before school…But for secondary students, they want to stay and hang around with their mates and friends from school, why not do it at school…it’s safer, there’s lots of areas to play, and they’re with their friends.*	✓	✓	✓	✓	✓
School attendance impacts	S11 (boy, secondary school): *They were trying to increase those numbers in the junior years where people weren’t coming to school on Friday mornings…It sort of encouraged them to come earlier, to get out of bed a bit earlier and get to school quicker. So yeah, I think it was successful.*	✓	✓			
Extension of school day for inclusive and safe engagement	Pr4 (primary and secondary school): *I don’t see it as babysitting. I think it’s an extension of the school day…But I also see co-curricular and I always stress it’s ‘co’, it’s not ‘extra’. It’s co-curricular because it’s part of the whole learning experience. And you look at the skills these students acquire as a result of their engagement [in physical activity programs].* E1: *In the north of here [Western Australia] and Queensland as well, you can’t do much physical activity at all…beyond 11 o’clock, it’s just too hot. So it’s a perfect time of day to be doing that.*	✓	✓	✓	✓	✓
Alignment with parent work schedules	Pa9 (primary and secondary school): *Having something to get them to, is just such a huge benefit…They’re the days I can get to an 8:30 meeting…rather than sort of push them out the car at a quarter to nine.*	✓	✓	✓	✓	✓
**Domain 2: Factors influencing before-school physical activity**						
***Individual***						
Attitudes, motivation, and enjoyment (+, −)	S7 (boy, secondary school): *My closest friends are all at like, the top level in football…They are really motivated to get better at the sport, and so they do stuff in the morning as well, just to get better. But I’d say for most people, it’s just like, “I don’t want to get up”.*	✓	✓	✓	✓	✓
Confidence and competence (+, −)	EP3: *Because it was focused on athletics, and it was sport-specific, you cut out a lot of children that may not necessarily feel confident or competent to run around an aths [athletics] track or to throw a javelin…The more confident ones stuck around, which is a common storyline, isn’t it?*	✓	✓	✓	✓	✓
Sleeping patterns and energy levels (+, −)	S8 (girl, primary school): *I think that it could be like a little bit later, instead of being like, so early, because you might not want to, like get up that early, or you could be a sleep-inner…it maybe could be like, maybe at least half an hour later. So that you actually have time to get ready for school, wake up, be ready to go and just not be tired.*	✓	✓	✓	✓	
Availability and use of time (+, −)	S6 (girl, secondary school): *I would never do anything else in the morning…I have training or work and stuff or homework in the afternoon to do. But usually in the morning, I don’t really do anything else…it’s just easy.*	✓	✓	✓	✓	
***Family and peers***						
Family home and work routines (+, −)	EP3: *Parents were really happy with the program, it was just, they were thinking it was too early. So it was busy in the morning, particularly primary school kids…You’ve got to get them up, you’ve got to organise them. And, suddenly there’s been homework that has not been completed that they told you that they did do and they didn’t do it. And so it’s a bit of a scramble. Then you’ve got parents going to work.*		✓	✓	✓	✓
Financial cost (+, −)	Pa10 (primary school): *There’s a cost to it each time as well. And I can just imagine, I’ve got one, but if you’ve got three kids, and they’re all doing something…*	✓	✓	✓	✓	
Parent involvement and support (+, −)	Pa3 (primary school): *I take my hat off to people…who are able to run these programs, but within my life, I don’t necessarily have the capacity to be contributing to those types of things frequently, maybe occasionally.*	✓	✓	✓	✓	✓
Transport to school (−)	T6 (secondary school): *Transport’s an issue…Some girls told us that “I just can’t get there because my parents can’t get me there"…They’d have to rely on public transport, school buses, to get there on time, which wasn’t possible.*	✓	✓	✓		
Breakfast provision (−)	Pa8 (primary and secondary school): *What can I do in terms of making sure…they have enough to eat or drink before the actual school day starts? And that’s always been a challenge…making sure that they’re full of energy, they’re not sort of depleted by the time morning tea comes around.*		✓			
Social influence (+)	S1 (boy, secondary school): *[There is] more time to meet up with friends, and you can have more time in the day to talk then, because I normally arrange with friends to go to school early.*	✓	✓	✓	✓	✓
Peer leadership (+)	T7 (primary school): *The big guys take the little guys around, and it’s just, it is lovely to see. And for some of the children, it is a highlight of their day…Sometimes it’s the naughtiest children that take the biggest pleasure out of showing something that they can do.*		✓	✓	✓	✓
***School***						
Availability of opportunities (+, −)	S9 (girl, primary school): *The only option is to like either go in the library and sit in air conditioning and like, read or go on the iPads or something, and only being able to play handball.*	✓	✓	✓	✓	✓
Access to facilities and equipment (+, −)	Pa7 (primary school): *What I would really love is just access to the playgrounds, for say, 15 min before the bell goes, so the kids can just run around and burn off some energy…Unfortunately, that’s not allowed. So the kids tend to run around in a space they’re not supposed to be running around in. And I don’t think that’s good for anyone, the teachers or the students, or parents.*	✓	✓	✓	✓	✓
Availability of staff supervision (+, −)	Pr4 (primary and secondary school): *[A barrier] is what it might place on your own teaching staff, because by and large, from a duty of care point of view, it’s ideal to have a teaching staff member on premises, and that’s a challenge…When you’ve got the right people, it work works well.* Pr2 (secondary school): *That girls gym program, at the moment that teacher does it all of her own, you know, passion, and the like…If programs like that work, though, again, I think if schools can look to resource that and support that teacher and/or the program…you can then support that program to keep running…Otherwise, relying on teachers’ goodwill, I find over time…they’ll have other priorities and things they need to move on to.*	✓	✓	✓	✓	✓
School priorities, timetabling and leadership support (+, −)	Pa2 (primary school): *So we did initially have a principal who was a little hesitant about it [a parent-run PA program]. But then we had a change of principal, and it was all systems go.* Pr2 (secondary school): *…there’s award agreements on teaching loads and things, but duties are generally up to principal’s discretion, so that we can supervise safely…It probably depends on the school and the priorities and how tight things are as to whether that’s [adding additional supervision duties] reasonable or not in different kind of school settings.*		✓	✓	✓	✓
Breakfast provision (+, −)	Pa1 (primary and secondary school): *I used to love going to cross country training…we would get for $1, a croissant, and a cup of fruit salad, which I just thought was fantastic. And that definitely helped me get out of bed as a teenager.*	✓	✓	✓	✓	✓
Incentives and rewards (+)	T1 (primary school): *It needs to be incentivised…We run prizes and things for people who run the furthest distance at ‘hundred kilometre club’…We’ve been lucky to get almost 100% participation for several years now.*	✓	✓	✓		✓
School size (−)	Pa1 (primary and secondary school): *I think the size of the school could be a barrier…With our school being so small…there’s going to be less availability for kids to actually turn up.*		✓	✓		
Uniform requirements (+, −)	E1: *For teenage girls in particular, uniform comes into it hugely…Having choice and some flexibility for girls in uniform has a huge impact on participating in those kinds of things too.*			✓		✓
***Community environment***						
Availability of opportunities (+, −)	EP2: *[In our before-school community family activity program] the parents can then get fit…It’s also a really great bonding time for parents to have with children, potentially the parent that goes off to work for a full day and isn’t home till after dinner time with children…So there’s some really great opportunities.*	✓	✓	✓	✓	✓
Parents and citizens associations (+, −)	Pa2 (primary school): *We [the P&C] began a subcommittee a few years ago, called the active living and sport subcommittee, because a few of us as parents were a little surprised when we got to school…expecting our children to have more sporting opportunities than what the school offered…We put a few proposals together for the school and a before school ‘kilometre club’ was one of those. And that was the key one that really, we were able to implement quite quickly, quite easily.*		✓	✓	✓	✓
Community partnerships and engagement (+)	Pr1 (secondary school): *One of the ways to maximise the success…is to involve the broader community…If you’ve got, ‘Mr. Smith’, who represented Australia, Queensland, New South Wales, at some sport at some level, and he’s involved with the school, either through the P and C, or not, he might be a key to that sustainability, because of his passion…It doesn’t matter what really happens with the staff at the school as they cycle through, the community drive it.*	✓	✓	✓	✓	✓
Safety (+,−)	Pa7 (primary school): *It feels dangerous to be riding bikes near the school…How do you encourage your children to actively transport to school if you don’t feel like that’s a safe choice?*		✓			
Accessibility to school (+, −)	Pr2 (secondary school): *Some schools are connected to train lines…we’re not, we’re quite a way from the train station, so if the kids aren’t within walking distance or riding distance it can be hard for them. They come from a fairly broad area to get to us…It’s very hard for them to either get here before school.*	✓	✓	✓	✓	
***Policy***						
Curriculum (−)	Pr1 (secondary school): *If it’s going to be important, we have to measure it. And what we’ve been asked to do by our systems…is that NAPLAN and the production of student results is far, far more important now than anything else, so measuring the performance of school sport becomes very problematic in schools and it gets put often in the too hard basket.*			✓		
Conditions and expectations of teacher employment (+, −)	Pr1 (secondary school): *The Catholic, independent and government sectors right across Australia have awards…they’ve got rules about how many teaching minutes they have…But that’s jurisdictionally based, so in Queensland, it’s different to New South Wales, it’s different to Victoria.*		✓	✓		
**Domain 3: Strategies for schools to support before-school physical activity**						
***Program design and implementation***						
Provide a range of context-specific activities	E2: *Type of activity becomes all important with the high school, given that flexible model of what they’re into…Gym-like stuff, or Pilates or something that kind of is not sport, but it’s a recreational, cool thing. And I like that idea of linking it in with something that’s of relevance.*	✓	✓	✓	✓	✓
Foster engagement and enjoyment	EP2: *Everything about our business and our program is very much play-based and fun, so that the kids don’t know that they’re essentially doing exercise or getting those minutes in that they need to. So for us, it’s about really employing the right coaches.*	✓	✓	✓	✓	✓
Make programs realistic, sustainable, and accessible	Pa2 (primary school): *Really key has to be sustainability…It’s great to promise the world to start with, but can we realistically keep that going every week?…That was a focus for us in just the ‘keep it simple’ philosophy. Because we needed to make it sustainable, that it didn’t require 10 volunteers, it only required two. And it didn’t require a lot of resources and a lot of different things, it only required one bucket’s worth of gear, and so that was easy for us to pass around for whoever was the coordinator for any given week.*	✓	✓	✓	✓	✓
Empower students through leadership and decision-making	E2: *…the student leadership group, meaning year six leaders or year twelves…just in terms of them helping deliver programs. And also, there’s also this link to that that house spirit, because most of them might be house leaders, and they can round up a bit of morale boosting stuff there around house competitions.*	✓	✓	✓	✓	✓
Provide participation incentives and recognition	Pa2 (primary school): *We wanted to give the kids a bit of incentive…We would give them a rubber band around their wrist to count their laps, and we would keep a tally of that…we did start to give prizes once they hit milestones…A little certificate or mentioned on assembly, when they were reaching the milestones.*	✓	✓	✓		✓
***Facilities and resources***						
Provide supervision	S8 (primary school): *At our school, a teacher comes out at 8:30 and they let us know that we can play handball. Maybe the teacher could like come out at like eight o’clock instead of 8:30, so if people arrive before 8:30, when a teacher comes at eight o’clock they can play instead of having to sit down and wait.*	✓	✓	✓		✓
Provide access to equipment and facilities	S6 (secondary school): *We have heaps of like ovals and basketball courts and stuff, but you’d have to bring your own ball. So maybe, like, just letting us borrow [the equipment].*	✓	✓	✓		
Provide access to food	Pr1(secondary school): *When I implemented a cricket and a rugby league program…we simply just had a conversation with the tuckshop or the canteen, and they opened a little bit early and provided additional food…It was very simple. It wasn’t a problem because the P and C ran the canteen, so it was just an easy thing to do.*	✓	✓	✓		✓
Provide additional transport options	T3 (secondary school): *How do we then offer an earlier bus route? If that was something that was holding students back from getting there. What sort of stakeholders need to be considered for that to happen?*			✓		
***Community engagement***						
Encourage community involvement and partnerships	T1 (primary school): *[Teachers and parents] like to come along and join in…We really encourage that, they can see the benefit it’s having for their kids, they can see their kids being out, fit and having fun with their friends before school…Those kinds of things make it as easy as possible for large numbers of people to be involved and make it as easy as possible for teachers to run those activities without creating this huge burden.*	✓	✓	✓	✓	✓
Engage parents and citizens associations	E1: *If you’ve got a health and wellbeing committee, that’s who you want on board. Sometimes if you have one champion, they leave and the whole thing falls apart. So it’s trying to engage a few people… When the burden of administering that program becomes too much, there’s other people that they can call on.*		✓		✓	✓
Communicate benefits to stakeholders	E1: *Having buy in, not only from the parents, but from the phys edder, from the principal, from classroom teachers is really important. And that can usually be done by selling other benefits…The kids have got rid of all that energy, that behaviour management is going to improve, and their early morning learning is going to improve because they’ve got all that out of their way, and they can concentrate a bit better.*		✓	✓	✓	✓
***Support for facilitators***						
Provide opportunities for training and development	T1 (primary school): *And that entailed going around to different regions and getting people together, talking to them about the importance of physical activity and getting buy-in from principals and from staff and from parents, and then giving a whole range of different ideas that have worked in other places…I think that kind of stuff really works…It made a huge impact. And there are still schools in our region, who continue those activities now, like our school, and many others, because of that initiative.*			✓		
Provide support and recognition	T5 (secondary school): *[When considering providing before-school opportunities] I always think, “well hang on, if I do that in the morning, and then go straight into a four-on day”, and then as a PE teacher, most of us will then have afternoon training or our own training afterwards…I get to 10pm before I get a break, starting early. So often, I’ll try and put it on days where you’ve got a spare period first…This year, just gone, I got taken off the home class line, which was amazing, because then I was putting lots on in the morning, because then even that 15 min of when everyone else is in home class is enough time…to reset and be ready for the day, as opposed to obviously all the equipment and packing up and everything that comes with being responsible for the space you’re in.*		✓	✓		

**Symbols**: + = influencing factor identified as a facilitator, − = influencing factor identified as a barrier; ✓ indicates that the corresponding subdomain was identified by the stakeholder group in the column.

**Stakeholder identifiers**: S = student, Pa = parent, T = teacher, Pr = principal, EP = external provider, E = school health and physical activity expert.

**Abbreviations**: NAPLAN = National Assessment Program– Literacy and Numeracy; P&C = school parents’ and citizens’ (otherwise known as parents’ and friends’) association.

### Domain 1: perceived value of before-school PA

In discussing their perceptions of before-school PA, stakeholders attached value to opportunities for children and adolescents to be active during this time. Several reasons for valuing before-school PA were identified, including perceived benefits for children and adolescents, their parents, and schools.

#### Extension of school day for inclusive and safe engagement in PA

Stakeholders described the before-school segment as an extension of the school day, creating additional PA opportunities outside of curriculum time. This was characterised as particularly beneficial for students less engaged in competitive sports or physical education, providing an inclusive opportunity to develop various skills. Stakeholders shared examples of programs established to capitalise on this opportunity. One program was designed for upper secondary school girls ‘to engage in something that was non-competitive, but an opportunity to engage in some exercise, and develop connections with people from across year levels’, (*Teacher 6*, secondary school) in response to observed decline in school sport involvement and the absence of compulsory health and physical education after year 10. An external provider also noted that these programs can offer broad development and cater to less active students:

*It was a handy selling point for them [the school], I think, because the programme was set up to include personal, social and emotional aspects, but also cross-curricular, and to give them more opportunity to move, which the parents didn’t necessarily have time to do. And a lot of these kids weren’t doing any other sport or physical activity anyway. So it was a good opportunity, outside curricular time, to be doing extra things.* (External Provider 3)

In regions with hotter climates, conducting activities before school was also perceived to take advantage of cooler temperatures and lower ultraviolet radiation levels, making it a safer and more comfortable time for students to be active. This was noted by stakeholders as a practical solution to climate-related challenges that restrict PA later in the day (see quote in Table 2).

#### Potential impact on school attendance

Stakeholders observed that before-school PA initiatives could enhance students’ motivation to attend school. One parent, recalling their child’s experience with a before-school PA program, said, ‘the day he does tennis…that day, it was like, “oh, no, I’m up, I’m here, I’m going”…which also confirmed for me that the other aspects [relating to claimed illness] were not necessarily an actual sore tummy, or the like’ (*Parent 9*, primary and secondary school). This parent also suggested that before-school programs may detach students from other issues causing resistance to attend school. Similarly, other parents shared how before-school programs positively influenced their children’s attitudes towards attending school, including one who observed ‘how excited, especially my middle child is, to be able to participate in this running program and getting certificates…He’s so excited to get to school on those days’ (*Parent 4*, primary school).

#### Potential impact on cognition, behaviour and wellbeing

Stakeholders perceived that engaging in PA before school could contribute meaningfully to students’ cognitive functioning, classroom behaviours, and wellbeing. Students, including those who admitted feeling tired upon waking, reflected on feelings of enhanced alertness and mental clarity after a bout of PA, which was also observed by school staff. Additionally, stakeholders discussed the role of PA in setting a positive mental disposition in students as they start their school day. One special education teacher particularly stressed the positive influence of before-school PA on students’ emotional regulation, highlighting its potential to ‘reduce stressors from possibly a morning that has been full of turmoil and getting [them] in a more relaxed, calm state’ (*Teacher 1*, primary school).

#### Potential impact on relationships

Before-school PA initiatives were identified as opportunities for social connection among students, often limited once formal class time begins. This was captured by one parent of a primary school student:

*If they’re coming in and just getting straight into the classroom, and everyone has to be quiet, there’s no talking. That [provision of PA before school] just might give the kids an opportunity to kind of catch up, talk about their weekend, welcome new kids in.* (Parent 6)

Important opportunities for social connection and rapport development between students and teachers were also noted. This idea was reinforced by a school PA expert who described the before-school segment as ‘a great time for teachers to connect with kids’, further noting that ‘you’re building relationships all the time when you’re doing those things before school’ (*Expert 2*).

#### Alignment with parent work schedules

Stakeholders indicated that before-school PA opportunities are convenient, often aligning with parent work schedules and providing a sense of security through supervision during hours when children might otherwise be unsupervised. One parent explained, ‘it’s quite a nice thing to be able to drop them off and know they’re going to be supervised and they’re doing something active’ (*Parent 6*, primary school). Parents also commented that PA programs in this segment have served as a form of before-school care, which is particularly beneficial for working parents who struggle to secure places in traditional before-school care services. Teachers noted that this appeal has had inadvertent benefits, leading to participation of children and adolescents who may not otherwise engage in PA. This was summarised by one participating school health expert:

*The appeal is actually to have kids supervised, it means that kids can be dropped off at school a bit earlier, and it’s going to be okay, they’re not breaking any rules. So that can actually be a benefit. It’s not the intended benefit, but it is, and sometimes gets kids who might not be active otherwise, there.* (Expert 1)

### Domain 2: factors influencing before-school PA

Factors perceived by stakeholders to be influential in enabling or limiting before-school PA spanned various levels of social-ecological influence, from the level of the individual (child or adolescent) to broader policy influence.

#### Individual level

Individual factors identified by stakeholders to influence before-school PA included psychological factors and aspects relating to the timing of activities. Confidence and competence were perceived as influential over children’s and adolescents’ decisions to participate and sustain their engagement in before-school PA opportunities. One student (*Student 7*, boy, secondary school) expressed concerns about participating in mixed-age environments before school, citing intimidation due to dominance of older students. Similarly, a principal (*Principal 2*, secondary school) highlighted the role of confidence and competence, noting that less physically active adolescents may be at higher risk of dropping out if they do not perceive improvement in physical skills.

Ranging sleeping patterns and energy levels also played a role in students’ preferences for participation in before-school activities. Some students described feeling fresh and preferring engaging in PA in the morning, while others found it challenging to wake up early. Teachers also acknowledged this dichotomy. They recounted instances where students voiced their desire for more opportunities to start their day with PA. However, they also recognised that adolescents, in particular, may have sleep patterns that do not align well with before-school PA, limiting participation of a number of students.

#### Family and peer level

Family and peer influences were identified, encompassing financial considerations, established family routines, parental involvement, transport challenges, and social interactions. Conflicting perspectives among parents were identified regarding the impact of morning routines on their children’s likelihood to participate in before-school PA. Some parents viewed the morning as highly opportune for additional activities, while others felt that time was limited. The following exchange between two parents demonstrates this:

*Sometimes you don’t want to be doing the after-school stuff, and then having to get them home and get dinner and get everything done…My friend calls morning time ‘dead time’ because…you wake up, [and] there’s so much time before you have to get to school.* (Parent 10, primary school)

*My family is the opposite…We always find it’s such a rush to get ourselves all ready, out the door, get the kids to school, get myself to work…I’ve got my children enrolled in a few after school activities, and that suits our schedule better. But I still see the benefit for them to be able to do something active before school for their focus, but how do I manage to fit the time in?* (Parent 7, primary school)

The time pressures faced by parents were also acknowledged by teachers, having heard concerns about the early timing of scheduled activities.

Stakeholders highlighted both parental support and transport-related issues as key influencing factors. Parental involvement was acknowledged for its positive impact on the successful implementation of before-school PA programs, as well as for reinforcing children’s and adolescents’ participation. Examples were recalled of parents demonstrating their interest by helping with before-school activities. However, stakeholders also recognised that parents’ availability and interest in PA could vary, potentially affecting their children’s opportunities to engage. Additionally, transport was identified as a major constraint, particularly for students relying on buses with infrequent schedules close to school start time. This was said to result in students partially or completely missing PA opportunities. The logistics of coordinating transport for multiple siblings also posed challenges and limited ability to participate in before-school PA.

#### School level

The widest range of influencing factors was identified at the school level, primarily centred around the allocation and availability of resources. According to stakeholders, a key factor influencing the before-school PA of children and adolescents is the extent to which schools offer structured opportunities for PA during these hours, including walking and running clubs, fitness programs, and organised sports training. Where structured programs were in place, stakeholders recalled instances of high student interest and participation rates, demonstrating the positive impact of these initiatives. The absence of such opportunities was a concern expressed by some stakeholders, who observed limited options and expressed a desire for PA in a segment with ‘untapped potential’ (*Expert 2*, school PA expert). In some circumstances, this lack of opportunities for students resulting in sedentary behaviours was the impetus for teachers to implement a program:

*I hate seeing kids at school sitting on concrete from 8:30 till 8:50, waiting for their names to be called on a roll. And it’s sort of been a bit of a personal mission to go ‘no, that that can’t happen, what can we be doing in that space?’ It’s such an amazing time to get kids switched on.* (Expert 2)

Outside of structured programs, access to school facilities and equipment was another influential factor determining participation in PA before school. Stakeholders described varied experiences regarding level of access in their respective schools. As an enabler of PA, it was noted that students use facilities such as basketball courts for before-school PA when they are open. However, instances of facilities or equipment being unavailable until later in the day were mentioned as deterrents to PA, with one student noting, “they don’t open the sports facilities in the morning, like at all, so you have to do it when a teacher’s there, so no one really can do it until later in the day.” (*Student 2*, girl, secondary school) Such limited access posed a challenge for students who relied on school-provided resources. Stakeholders also described school policies that limited access to facilities, restricting students from using playgrounds or sports equipment, and often resulting in sedentary behaviours, before school.

Availability of staff supervision was widely discussed for its crucial role in determining whether PA programs or facilities were available before school, with PA being discouraged until staff were available to supervise. Stakeholders acknowledged the importance of supervision in minimising risks and liabilities but noted the complexity of factors influencing staff availability, including teachers’ goodwill, expertise and experience, increasing workload, and competing school priorities. Various stakeholder groups acknowledged that teachers’ busy schedules (within and outside work) and the demands of their teaching responsibilities often hindered their ability to dedicate time to extracurricular offerings. Staff turnover, particularly when programs relied on a sole teacher as leader, was identified as a challenge for program sustainability, often reflected in program transience. Indeed, specific instances were recalled where the departure of the key teacher or their newfound involvement in other commitments led to program discontinuation.

#### Community environment level

At the level of the community environment, stakeholders discussed the influence of several factors on before-school PA, including the role of school parents’ and citizens’ (P&C) associations. P&C associations were recognised for their substantial influence in schools, including as advocates for health and wellbeing initiatives. Stakeholders recalled various instances in which P&C associations were seen as pivotal in driving volunteer-led or externally provided before-school PA programs. Reflecting on P&C-championed programs (largely facilitated by parent and community volunteers), community involvement was perceived to help alleviate the burden on schools with limited resources and capacity to offer activities, supporting the sustainability of programs over time. However, stakeholders also acknowledged the challenges associated with recruiting adequate numbers of volunteers, attributed to their busy work lives and other commitments.

In addition to the influence of P&C associations and community volunteers, examples were identified of community members and organisations partnering with schools in support of before-school PA. These community partnerships involved the provision of resources, including donations of awards for students, and were said to contribute to program sustainability and extend the reach of the initiatives.

Relating to the physical environment, stakeholders identified safety concerns and accessibility to schools as factors shaping children’s and adolescents’ mode of transport to school and, consequently, their engagement in before-school PA. Safety concerns related to heavy traffic around schools were highlighted by parents, leading them to accompany their children when using active transport modes, or to instead use passive transport. Additionally, several factors impacting accessibility to schools were perceived to influence the likelihood of engaging in active transport or school-based PA before school. These factors included the physical characteristics of the environment (e.g., terrain), the proximity of public transport options, and the distance between home and school. In some rural communities and small towns, favourable conditions for before-school PA were observed due to shorter distances and limited reliance on motorised transport. Conversely, stakeholders indicated that urban areas posed greater challenges in this regard.

#### Policy level

At the government policy level, stakeholders highlighted two influencing factors: curriculum pressures and teacher employment conditions. Employment agreements, policies governing school systems, and competing curriculum priorities were raised as complex factors that influence before-school PA at school, primarily by way of guiding the allocation and availability of staff supervision. While supervision challenges and decisions were reported earlier at the level of the immediate school environment, where they were primarily said to be managed, these upstream factors were also identified as influential. Principals provided examples of industrial agreements that prevent timetabling teachers for before-school programs unless they receive time off in lieu. Therefore, voluntary participation of teachers to support these programs was acknowledged as a common occurrence. Variations in policies were noted between states and school systems. For example, different awards and an identified expectation for staff engagement with co-curricular aspects of schooling in the independent sector was perceived by some stakeholders as a facilitating factor. Additionally, stakeholders identified the low policy status of school PA compared to curriculum priorities as a challenge for the allocation of resources and implementation of programs, despite the separation of the before-school segment from curriculum time.

### Domain 3: strategies for schools to support before-school PA

Stakeholders highlighted strategies across four key categories: program design and implementation, facilities and resources, community engagement, and support for facilitators. In the category of program design and implementation, stakeholders suggested providing a variety of activities specific to the school context, and involving students in decision-making regarding the PA initiative. Stakeholders proposed improvements to facilities and resources, including open access to sporting equipment, as well as availability of breakfast options, showers, and changing facilities. Strategies for community engagement included engaging school P&C associations and establishing partnerships with external organisations. Finally, stakeholders highlighted the importance of ensuring the individuals facilitating before-school PA initiatives are appropriately supported, by providing training, recognition, and in the case of school staff, timetabling support. These strategies are presented in full in Table 2.

## Discussion

Despite recommendations for schools to support before-school PA [6], key stakeholders’ perspectives on its value, feasibility and effective implementation have received limited research attention. This study addresses this gap by qualitatively exploring stakeholder perspectives on before-school PA, its influencing factors, and potential strategies to support initiatives in schools. The results highlight the high value stakeholders place on before-school PA, attributed to a range of perceived benefits for students, parents, and schools. Varied insights from stakeholders—students, teachers, parents, school leaders, external providers, and experts—provide a comprehensive picture of the complex, multi-level social-ecological factors that influence engagement in before-school PA. Stakeholders also highlighted sustainability challenges, often due to program dependence on individual teachers for leadership and facilitation. In response, they proposed school-based strategies across various categories, including program design and community engagement, aiming to enhance the sustainability and participation in before-school PA initiatives. These findings offer practical implications for schools intending to strengthen before-school PA opportunities and provide a foundation for developing effective interventions and resources tailored to this context.

Stakeholders overwhelmingly cited perceived learning-related benefits as a primary reason behind the importance of before-school PA for children and adolescents. Interestingly, physical health benefits and direct impacts on PA levels were rarely mentioned. This suggests that stakeholders consider these aspects inherent to PA participation across the board, focusing more on the immediate relevance of cognitive impacts in the before-school setting. In recent times, there has been an increase in studies investigating the effects of before-school PA programs [9], with several studies focusing on learning-related outcomes, such as on-task behaviour [11] and concentration [10, 29]. While current evidence is limited, a positive association has been identified between before-school PA and readiness to learn [9]. This finding aligns with stakeholders’ beliefs that morning PA can improve factors such as on-task behaviour and reduce daytime sleepiness. Although further empirical research is needed to improve understanding of the relationship between before-school PA and aspects relevant to student learning, the preliminary evidence and stakeholder perspectives gathered in this study suggest that highlighting cognitive benefits might be an effective way to encourage stakeholder ‘buy-in’. This approach may be combined with additional evidence on the potential social-emotional wellbeing benefits of before-school PA [30], which were also discussed by stakeholders in this study.

Findings from this study emphasise the importance of the school setting in shaping before-school PA. Many factors identified by stakeholders relate closely to this level of the social-ecological model, indicating its substantial impact. These findings provide insight into the disparities in the prevalence of PA-supportive practices in the before-school segment compared to during and after school [31–33]. Key issues, such as the availability of facilities for free play or provision of structured programs before school, were acknowledged for their complex influences upon PA. The availability of qualified supervision was identified as necessary for schools to support before-school PA, which in turn was said to be determined by upstream factors including school leadership support and policies regarding teachers’ workloads and work hours. While stakeholders expressed frustration at school rules prohibiting before-school PA, they acknowledged these policies often stem from concerns about risks and liabilities associated with unsupervised students engaging in PA. Although regular supervision before school was noted to be atypical, principals suggested that with leadership support, appropriate supervision resources could be mobilised if PA initiatives were aligned with their priorities. Previous studies have highlighted this influential role of school leadership in the success of school PA initiatives [34, 35]. Future research is required to investigate the impact of before-school policies and practices on children and adolescents’ PA levels.

Participants noted a common pattern of transient before-school PA programs, often attributed to their reliance on a sole, goodwill-driven teacher who championed and facilitated these initiatives. Indeed, previous research has identified a similar challenge where the successful implementation of PA programs often requires ‘personal initiative, planning, and investment’ from teachers or administrators, who are not compensated for this extra work [36]. Stakeholders in our study described instances of program discontinuation following the departure of such pivotal teachers or changes in their commitments. This highlights the limitations of programs reliant on an individual champion, and aligns with previous research on the vulnerability of such approaches to program sustainability [37, 38]. An embedded, systems-based approach which promotes collaborative efforts could offer a more sustainable and effective solution, aligning with calls for such frameworks in the wider field of PA promotion [39]. Engaging external providers is a potentially promising strategy, although stakeholders noted financial burdens on families due to this approach. Moreover, this practice of outsourcing extracurricular PA programming has been highlighted as requiring critical exploration and consideration of potential unintended consequences [40]. 

While volunteer-driven or supported initiatives offer some solutions, stakeholders noted their success remains precarious as they are often contingent on individual commitment. *Build Our Kids Success* is an example of a before-school program delivered by trained volunteers, including parents [41], which has seen extensive adoption by schools since its inception in the United States [42]. However, stakeholders in our study identified challenges in recruiting adequate volunteers and proposed potential solutions, like implementing simple programs with a reduced load and providing appropriate training. Separate research suggests that providing incentives could further strengthen outcomes [43]. Rather than relying solely on individual or volunteer efforts, a systems-led approach could offer more robust and sustainable solutions by facilitating cross-sector collaboration and efficient resource use [39]. For instance, lessons can be drawn from research examining university-school partnerships where pre-service teachers implement before-school PA programs under a structured system [44, 45]. In addition, the influence of P&C associations at the community level was noted by stakeholders due to their advocacy, direct facilitation of programs, and existing community partnerships. Accordingly, P&C associations and community partnerships could be mobilised within this systems-based framework to offer long-term solutions that are embedded within the school community.

Although the potential benefits of before-school PA were appreciated by most stakeholders, we found perceptions of feasibility regarding the morning hours to be divisive among parents and young people. For instance, while some parents and students felt they had sufficient time available in the morning for additional activities, others saw the appeal of before-school PA but struggled to reconcile this with an already busy morning routine. Indeed, a recent cross-sectional study using time-use diaries found that 56% of adolescents did not report engaging in PA before school, and among those who did, most was transport-related [46]. Barriers of time availability extend beyond the before-school segment. One study identified lack of time as a barrier for 10–13 year-old children participating in after-school PA, including organised and non-organised PA and active transport [47]. After-school time restrictions might largely relate to students’ competing commitments, such as homework [47]. In contrast, we found that before-school time constraints may be more closely linked with sleep patterns and morning preparations, although stakeholders did recognise competing opportunities such as music lessons. A study examining the preferences of US parents (with children in elementary school) for before- and after-school PA program attributes found a preference for after-school programs [48]. Furthermore, parents prioritised programs that developed skills rather than free play [48]. However, these preferences may differ across regions and school communities, and, as acknowledged by the authors, parental preferences may not translate to their children’s attendance [48]. Given these variable preferences and contexts, providing a range of opportunities may cater to different needs, schedules, and preferences, including the before-school segment. Understanding the factors that motivate students to engage consistently across different opportunities will be crucial in designing effective programs.

Stakeholders highlighted a range of strategies for schools to support before-school PA. Among these was to provide context-specific activities, and to include students in the decision-making process through co-design initiatives. Stakeholders recognised the importance of the type of activity, highlighted as particularly critical for secondary school students, and that this should align with their interests. This resonates with a study that identified the importance of PA clubs offering culturally relevant and popular physical activities appreciated by students as interesting and meaningful outside of school [35]. Additionally, the opportunity to participate in ‘individual, non-competitive, non-sport physical activities’ was reported as appealing for students and a point of contrast from the activities they had experienced in physical education [35]. This is consistent with the experiences shared by school personnel in our study who adapted to their students’ preferences by providing non-competitive opportunities before school.

Another strategy identified to achieve buy-in was the effective communication of before-school PA benefits to stakeholders. To ‘sell’ these benefits effectively, stakeholders noted that connections can be made to school improvement priorities, such as student wellbeing or attendance, which were perceived benefits noted in our results. Although our study indicated that before-school PA is valued by stakeholders, this sentiment may not be universal. Therefore, it may be necessary to raise awareness about the added value that PA in this segment can bring to schools by ‘speaking their language’ [49]. However, we acknowledge that valuing PA alone may not suffice to enhance opportunities, and sustainable models of supervision will be necessary to ensure the longevity of such programs.

This is the first qualitative study to examine multiple stakeholder perspectives on before-school PA beyond specific programs. A major strength is the inclusion of a diverse range of stakeholder groups, enabling the identification of influencing factors and strategies from multiple perspectives and across multiple social-ecological levels. This approach not only aligns with our subtle realist paradigm, acknowledging the influence of varied experiences and assumptions on the representation of phenomena, but also offers a broad perspective on issues that may influence future interventions. The trustworthiness of our findings is enhanced by robust measures such as independent coding of data and maintenance of an audit trail of coding template iterations.

We acknowledge some limitations of the study. Our sampling strategy led to a sample of stakeholders who are rather highly engaged with school PA, including an overrepresentation of health and physical education teachers. As a result, the perspectives of other individuals, including teachers, students and parents, who are not involved in these activities are not captured. While our study captured the perspectives of stakeholders who have experience with PA for children of various age groups, it is important to note that direct input from younger children was not included. Further, the exclusive participation of women in the parents’ focus groups may not fully represent the perspectives of all parents. Future studies may benefit from engaging a wider variety of stakeholders to ensure more diverse representation. Considering data collection methods, focus groups were employed to facilitate group interaction. However, these interactions might have been limited by the small participant numbers in some sessions, attributed to participant availability and absences, with one session reverting to an individual interview. Regarding data analysis, we employed template analysis, which allowed for the description of a comprehensive range of influencing factors and strategies. While this technique supported our aims, future research may call for an analysis approach that allows greater interpretative analysis to more deeply examine complexities identified by stakeholders.

## Conclusions

This study provides insight into diverse stakeholder perspectives on before-school PA, its influencing factors, and strategies to support before-school PA programming. The perspectives shared by stakeholders, including students, teachers, parents, school leaders, external providers, and experts, highlight the complexity of the subject and the varied factors that can affect the provision of and participation in PA before school. In addition, these findings demonstrate the value attributed to before-school PA by stakeholders, largely for perceived cognitive benefits, and its potential to enrich school environments. With these insights, this research offers a valuable foundation for schools seeking to enhance opportunities for PA before school hours. Its findings may also inform future research examining the impacts of before-school PA initiatives.

### Electronic supplementary material

Below is the link to the electronic supplementary material.

Supplementary Material 1

Supplementary Material 2

Supplementary Material 3

## Data Availability

Summarised data analysed for this study are included within the text and tables of this article.

## Abbreviations

ICSEA: Index of Community Socio-Educational Advantage
PA: Physical activity
P&C: Parents’ and citizens’ (association)
