# Supplementary material for: A qualitative exploration of multi-stakeholder perspectives of before-school physical activity
Source: Int J Behav Nutr Phys Act. 2024 Feb 29;21:25. doi: 10.1186/s12966-024-01572-z (PMC10905879; doi:10.1186/s12966-024-01572-z)
Supplement: Supplementary file 1 — Supplementary Material 1 [file 12966_2024_1572_MOESM1_ESM.docx]

| **Supplementary table 1.** Focus group questions | | | |
| --- | --- | --- | --- |
|  | **Students** | **Parents** | **Teachers, external providers, experts** |
| *Introduction to session* | The moderators introduced themselves and the study aims. Participants verbally confirmed consent and agreed to session recording. | | |
| *Introduction to participants* | Please introduce yourself by name, grade, and school start time.  What is your experience with physical activity before school? | Please introduce yourself by name and in the context of before-school physical activity (i.e., if you have experience, in what program and what capacity?). | |
| *General opening question* | What are your thoughts about the physical activities you have participated in before school? What has made you participate? | What are your thoughts about before-school physical activity opportunities?  Why have you supported (or not) your child to participate in before-school physical activity? | What are your thoughts about before-school physical activity opportunities?  Why have you / your school supported (or not) before-school physical activity? |
| *Open-ended questions – influencing factors* | What things make it easy (or help you) to be active before school?  What things make it difficult to be active before school? | What enabling factors assist your child to be active before school?  What barriers make it hard for your child to be active before school? | What enabling factors assist students to be active before school?  What barriers are associated with before-school physical activity? |
| *Open-ended questions – strategies* | What would help (or has helped) you to participate in before-school physical activity?  In what ways could schools help this? | What would help (or has helped) to support your child’s participation in before-school physical activity opportunities? | What would help (or has helped) schools or school personnel to facilitate or support before-school physical activity? |
| *Summing up* | Participants were asked if they have further questions, additions to previous answers, and suggestions for questions to ask future groups. | | |
