# Supplementary material for: A qualitative exploration of multi-stakeholder perspectives of before-school physical activity
Source: Int J Behav Nutr Phys Act. 2024 Feb 29;21:25. doi: 10.1186/s12966-024-01572-z (PMC10905879; doi:10.1186/s12966-024-01572-z)
Supplement: Supplementary file 2 — Supplementary Material 2 [file 12966_2024_1572_MOESM2_ESM.docx]

| **Supplementary table 2.** Definitions used for coding influencing factors to social-ecological levels | |
| --- | --- |
| Level | Definition |
| Individual | Factors that relate to the individual student, including their knowledge, attitudes, motivations and perceived competence in physical activity. Factors at this level may include personal preferences for, or enjoyment of, different types of physical activity and individual decisions regarding time use. |
| Family | Factors that relate to the student’s family, including family support, family involvement in physical activity, and socioeconomic status. Factors at this level may be determined by availability of resources (such as time and money) for physical activity. |
| Peers | Factors that relate to the student's social environment, including peer support and influence, and social norms related to physical activity. |
| School | Factors that relate to the school environment, both physical and organisational. This includes the availability of physical activity facilities, opportunities for physical activity, and the attitudes, practices, and expertise of school staff. |
| Community environment | Factors that relate to the student's broader community environment, including the availability and accessibility of community programs and resources for physical activity, and the social and cultural position of the community, as well as conditions of the physical and built environment within the community affecting physical activity. |
| Policy | Factors that relate to government policies and regulations that affect physical activity, such as policies of state education systems and the allocation of resources to support schools. |
