# Supplementary material for: A qualitative exploration of multi-stakeholder perspectives of before-school physical activity
Source: Int J Behav Nutr Phys Act. 2024 Feb 29;21:25. doi: 10.1186/s12966-024-01572-z (PMC10905879; doi:10.1186/s12966-024-01572-z)
Supplement: Supplementary file 3 — Supplementary Material 3 [file 12966_2024_1572_MOESM3_ESM.docx]

| **Supplementary table 3.** Final coding template |
| --- |
| **1. Perceived value of before-school physical activity** |
| *1.1 Cognitive, behavioural and wellbeing impacts* |
| *1.2 Relationship impacts* |
| *1.3 School attendance impacts* |
| *1.4 Extension of school day for inclusive and safe engagement* |
| *1.5 Alignment with parent work schedules* |
| **2. Factors influencing before-school physical activity** |
| *2.1 Individual* |
| 2.1.1 Attitudes, motivation, and enjoyment |
| 2.1.2 Confidence and competence |
| 2.1.3 Sleeping patterns and energy levels |
| 2.1.4 Availability and use of time |
| *2.2 Family and peers* |
| 2.2.1 Family home and work routines |
| 2.2.2 Financial cost |
| 2.2.3 Parent involvement and support |
| 2.2.4 Transport to school |
| 2.2.5 Breakfast provision |
| 2.2.6 Social influence |
| 2.2.7 Peer leadership |
| *2.3 School* |
| 2.3.1 Availability of opportunities |
| 2.3.2 Access to facilities and equipment |
| 2.3.2.1 Availability of equipment for PA |
| 2.3.2.2 Availability of facilities for PA |
| 2.3.2.3 Availability of changing and showering facilities |
| 2.3.2.4 Risks and liabilities |
| 2.3.3 Availability of staff supervision |
| 2.3.3.1 Expertise, training and personal interests |
| 2.3.3.2 Teacher turnover |
| 2.3.3.3 Workload and competing priorities |
| 2.3.3.4 Teachers’ goodwill |
| 2.3.4 School priorities, timetabling and leadership support |
| 2.3.5 Breakfast provision |
| 2.3.6 Incentives and rewards |
| 2.3.7 School size |
| 2.3.8 Uniform requirements |
| *2.4 Community environment* |
| 2.4.1 Availability of opportunities in the community |
| 2.4.2 Parents and citizens associations |
| 2.4.3 Community partnerships and engagement |
| 2.4.4 Safety |
| 2.4.5 Accessibility to school |
| *2.5 Policy* |
| 2.5.1 Curriculum |
| 2.5.2 Conditions and expectations of teacher employment |
| **3. Strategies to support before-school physical activity** |
| *3.1 Program design and implementation* |
| 3.1.1 Provide a range of context-specific activities |
| 3.1.1.1 Align activities to the specific school context |
| 3.1.1.2 Provide a range of activities |
| 3.1.1.3 Provide age-specific opportunities |
| 3.1.1.4 Adapt activities to available facilities |
| 3.1.2 Foster engagement and enjoyment |
| 3.1.2.1 Establish a welcoming and engaging environment |
| 3.1.2.2 Provide fun activities |
| 3.1.3 Make programs realistic, sustainable, and accessible |
| 3.1.3.1 Purposefully determine start time |
| 3.1.3.2 Provide activities across different days |
| 3.1.3.3 Use minimal resources |
| 3.1.3.4 Set realistic timeframes |
| 3.1.3.5 Provide free-of-charge opportunities |
| 3.1.4 Empower students through leadership and decision-making |
| 3.1.4.1 Harness peer leadership |
| 3.1.4.2 Solicit for student voice |
| 3.1.5 Provide participation incentives and recognition |
| *3.2 Facilities and resources* |
| 3.2.1 Provide supervision |
| 3.2.2 Provide access to equipment and facilities |
| 3.2.2.1 Provide access to PA facilities |
| 3.2.2.2 Provide access to PA equipment |
| 3.2.2.3 Provide access to changing and showering facilities |
| 3.2.3 Provide access to food |
| 3.2.4 Provide additional transport options |
| *3.3 Community engagement* |
| 3.3.1 Encourage community involvement and partnerships |
| 3.3.2 Engage parents and citizens associations |
| 3.3.3 Communicate benefits to stakeholders |
| 3.3.3.1 Make connections to measurable outcomes and school priorities |
| 3.3.3.2 ‘Sell’ the benefits |
| *3.4 Support for facilitators* |
| 3.4.1 Provide opportunities for training and development |
| 3.4.1.1 Provide training to staff |
| 3.4.1.2 Share ideas between schools |
| 3.4.2 Provide support and recognition |
| 3.4.2.1 Support staff through timetabling |
| 3.4.2.2 Establish program roster to ease workload of facilitator |
| 3.4.2.3 Provide facilitator with prepared plans |
| 3.4.2.4 Provide recognition to staff |
